# Characterization dataset of oil palm empty fruit bunch (OPEFB) fibers – Natural reinforcement/filler for materials development

**DOI:** 10.1016/j.dib.2022.108618

**Published:** 2022-09-17

**Authors:** Alex Darío Aguilar, Vladimir Valle, Cristina E. Almeida-Naranjo, Ángel Naranjo, Francisco Cadena, Jerónimo Kreiker, Belén Raggiotti

**Affiliations:** a Departamento de Ciencias de Alimentos y Biotecnología, Escuela Politécnica Nacional, Ladrón de Guevara E11-253, Quito, Ecuador; b Universidad de las Américas, Redondel del Ciclista Antigua Vía a Nayón, Quito, Ecuador; c Department of Mechanical and Materials Engineering, University of Nebraska, Lincoln, NE 68588, United States; d Departamento de Matemática, Escuela Politécnica Nacional, Ladrón de Guevara E11 - 253, Quito, Ecuador; e Centro Experimental de la Vivienda Económica (CEVE)-CONICET, AVE. Igualdad 3585, Córdoba, Argentina; f Centro de Investigación, Desarrollo y Transferencia de Materiales y Calidad (CINTEMAC), UTN-FRC, Maestro M. López y Cruz Roja Argentina, Córdoba, Argentina

**Keywords:** Natural fiber properties, Statistical function for fiber length, Testing method for fiber length, Thermal degradation, FTIR, Mechanical properties, OPEFB, Oil palm empty fruit bunch, MLE, Maximum likelihood estimation, KS, Kolmogorov-Smirnov

## Abstract

Natural fibers used as reinforcements or fillers for materials development greatly affect properties and performance of end-use applications. As a consequence of conditioning processes such as grinding and sieving, average fiber length varies significantly. It is thus necessary to estimate the length as statistical data distribution rather than a single mean value. This approach implies length measurement of a significant number of fibers; however, a very high number of data points requires not only long-time frames but also significative amount of work. To address these issues, this article details a facile methodology to measure the length of a large number of natural fibers of oil palm empty fruit bunch (OPEFB) together with a statistical analysis to verify the correspondence between theoretical distributions and experimental data. Moreover, further information related to spectrophotometric, physico-chemical, mechanical, thermal, and morphological characteristics of OPEFB fibers coming from oil palm cultivation in Ecuador are presented. The data will contribute to comprehensively and rigorously describe the overall effects of natural fiber lengths on material properties.

## Specifications Table

**Table:** 

Subject:	Material Characterization
Specific subject area:	Agro-industrial waste valorization, Natural fibers
Type of data:	Tables, Images, and Figures
How the data were acquired:	Oil palm empty fruit bunch (OPEFB) fiber length data were acquired through image processing with ImageJ® software. Python library SciPy® and Python package distfit® were used to analyze the data and determine the OPEFB fiber length distributions. Infrared spectrophotometer, thermobalance, universal testing machine, and scanning electron microscope were employed to obtained physico-chemical and morphological characteristics of OPEFB fibers.
Data format:	Raw and processed data
Description of data collection:	OPEFB waste was milled and sieved to obtain different fiber length groups. Images of each group were captured keeping focal length (5.58 mm), aperture (f/1.8), sensitivity (ISO 100), and resolution (4608×3456 pixels) constant. The scale was established with a standard precision graduated ruler. About 40 images per length group were processed. ImageJ® software was used for the measurement of the fiber length. Analytical methods according to ASTM standards were used to quantify chemical components of OPEFB fibers. Instrumental techniques, i.e., infrared spectroscopy, thermogravimetric analysis, and electron microscopy were applied to OPEFB fibers without further preparation.
Data source location:	OPEFB samples were collected at: *Institution:* Teobroma-Alcopalma industry *City/Country:* Quinindé, Ecuador *Latitude and longitude:* 0° 20′N 79° 29′W Data was processed at: *Institution:*Centro de Investigaciones Aplicadas a Polímeros (CIAP), Escuela Politécnica Nacional *City/Country:* Quito, Ecuador
Data accessibility:	Repository name: Mendeley Data Data identification number: doi: 10.17632/4y32ks2t62.2 Direct URL to data: https://data.mendeley.com/datasets/4y32ks2t62/2

## Value of the Data

• The testing method for fiber length can be used by researchers to measure the dimensions of a large number of fibers. Statistical analysis of OPEFB fiber length data led to establish models that can be applied to other natural fibers.
• Fiber length distribution after milling process is a particularly important parameter in natural fiber reinforced composite materials, adsorbents in batch/continuous adsorption processes, and as briquettes for power generation.
• The characterization data provide valuable information on the physico-chemical, morphological, and dimensional properties of OPEFB fibers from biowaste widely generated worldwide.

## 1 Data Description

The non-homogenous nature of OPEFB fibers has paramount influence on properties and performance of end-use applications [1,2]. In this regard, average fiber length varies markedly, not only in OPEFB fibers, but also in a great number of natural fibers, which magnifies and diversifies the aforementioned influence [3,4]. Particularly, as a result of conditioning processes such as grinding, the fiber length dispersion increases considerably. In the case of natural fiber sieving, the retention is not solely determined by sieve aperture size. The heterogenous dimensions of fibers, superficial defects, static electricity, and so on combined by random movement during the process leading to a very complex mechanism of classification. In this context, it is necessary to characterize the length as a range or distribution rather than a single mean value. However, getting numerous measurements of fiber length in shorter time frames is difficult to achieve due to the amount of work required.

**Fig. 1:**
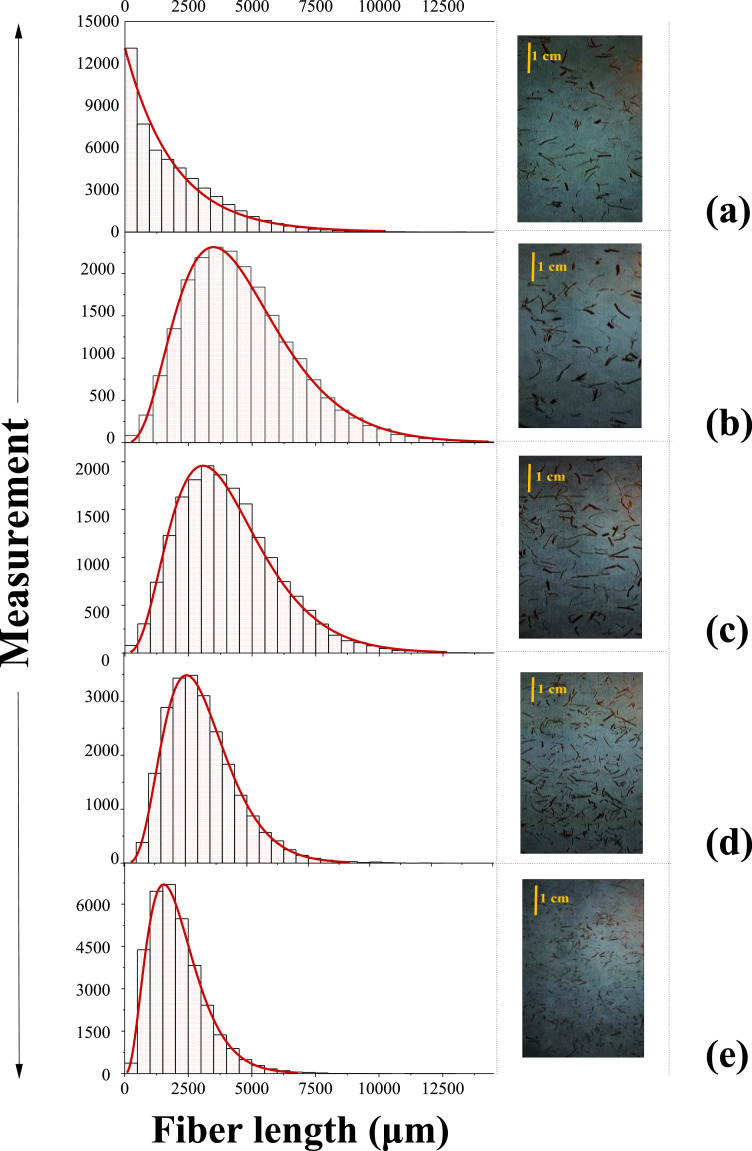
Images and length distribution of non-sieved OPEFB fibers (a) and OPEFB fibers retained in meshes No. 20 (b), 30 (c), 40 (d), and 50 (e).

On the other hand, it is also important to complement information regarding main properties of OPEFB fibers coming from different geographical zones worldwide. Since physical, chemical, and morphological properties of oil palm cultivation may change depending on edaphoclimatic conditions, further characteristics are essential to describe and contrast the effects of using their wastes on end-use applications. In this article, raw OPEFB fibers coming from oil palm cultivation in Ecuador were evaluated to obtain reference data on their length distribution, functional groups, as well as on their chemical, mechanical, thermal, and morphological properties. Heterogeneity of non-sieved OPEFB fibers, as well as of OPEFB fibers retained on meshes No. 20, 30, 40, and 50 can be seen in Fig. 1, while fiber length data are available at Mendeley data repository (Link: https://data.mendeley.com/datasets/4y32ks2t62/2) [5]. Additionally, summary statistics of fiber length from each group studied are shown in Table 1.

**Table 1:** Summary statistics of the OPEFB fiber length from each group studied.

OPEFB Fibers retained on mesh No.	Number of Observations	Minimum (µm)	Maximum (µm)	Mean (µm)	Standard Deviation (µm)
non-sieved	53,324	37.02	46,304.93	1,930.38	1,766.41
20	21,514	202.90	21,675.63	4,591.10	2,223.19
30	17,822	207.56	16,992.15	4,052.44	1,935.03
40	22,981	255.00	12,518.86	3,177.98	1,450.93
50	33,087	202.75	12,570.12	2,092.85	1,119.79

A theoretical distribution was determined for each data set in order to characterize the fiber length, as can be seen in Fig. 1. The non-sieved fiber length data fitted an exponential distribution (Eq. (1)):

(1) $$f\left(l\right)=\frac{1}{\theta }e^{-\frac{l-l_{0}}{\theta }}$$

where l is the OPEFB fiber length, and $l_{0}$ and θ are the location and scale parameters of the exponential distribution, respectively.

Meanwhile, the length data of OPEFB fibers retained on meshes No. 20, 30, 40, and 50 fitted three-parameter gamma distributions (Eq. (2)):

(2) $$f\left(l\right)=\frac{l^{\left(k-1\right)}}{\Gamma \left(k\right)\theta^{k}}e^{-\;\frac{l-l_{0}}{\theta }}$$

where l is the OPEFB fiber length; $l_{0}$, θ, and k, are the location, scale, and shape parameters of the gamma distribution, respectively, and Γ(k) is the gamma function (Eq. (3)). The parameters of the gamma distribution are related to the mean μ and variance $\sigma^{2}$ by the Eq (4) and Eq (5), respectively.

(3) $$\Gamma \left(k\right)=\int_{0}^{\infty }x^{k-1}e^{-x}$$

(4) $$\mu =k\theta +l_{0}$$

(5) $$\sigma^{2}=k\theta^{2}$$

Besides, the parameters of the theoretical fitted distributions for each group studied of the OPEFB fiber length, their standard errors for the maximum likelihood estimation (MLE), and p-value for Kolmogorov-Smirnov (KS) test are presented in Table 2.

**Table 2:** Parameters of theoretical fitted distributions for OPEFB fiber length data, standard errors for MLE, and *p*-value for KS test.

				Estimated errors			

OPEFB Fibers retained on mesh No.	$l_{0}$	θ	k	$\sigma_{l_{0}\;}$	$\sigma_{\theta \;}$	$\sigma_{k\;}$	*p*-value (KS)
non-sieved	37.021	1893.360	-	0.036	8.200	-	<0.0010
20	-432.463	976.185	5.146	55.821	17.342	0.142	0.6190
30	-576.113	815.505	5.676	62.904	16.814	0.186	0.0268
40	-96.561	608.368	5.393	37.171	10.736	0.150	0.0038
50	199.624	646.740	2.927	7.079	6.681	0.037	0.1076

**Fig. 2:**
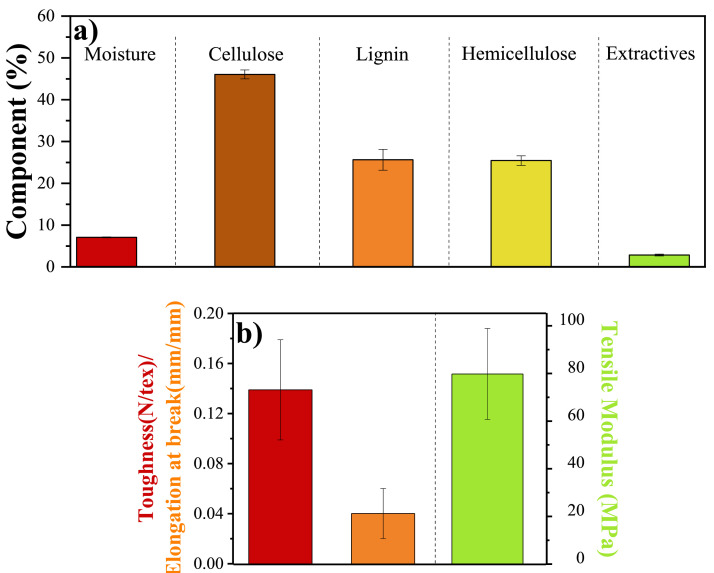
(a) Physico-chemical and (b) mechanical properties of OPEFB fibers.

On the other hand, the physico-chemical and mechanical properties are illustrated in Fig. 2a. and 2b., respectively. It was determined that lignocellulosic materials (lignin, cellulose, and hemicellulose) were the main components of the fibers, with the cellulose content predominating.

Furthermore, Table 3 shows the data from Fourier transform infrared (FTIR) spectroscopy, where the wavelengths of the most important bands and the functional groups to which they were associated are described. These functional groups were present in cellulose, hemicellulose, and lignin.

**Table 3:** Functional groups of OPEFB fibers.

Wavenumber (cm^−1^)	Associated functional group [6,7]
3332	O-H stretching vibrations
2920	C-H stretching vibrations of methyl and methoxy groups
1730	C=O stretching vibration of acetyl groups
1591	C=O stretching vibration of carboxyl and carbonyl groups
1023-1317	C-O stretching vibration of carboxylic acids, alcohols, phenols, ethers, and/or esters
552	aromatic structure

**Fig. 3:**
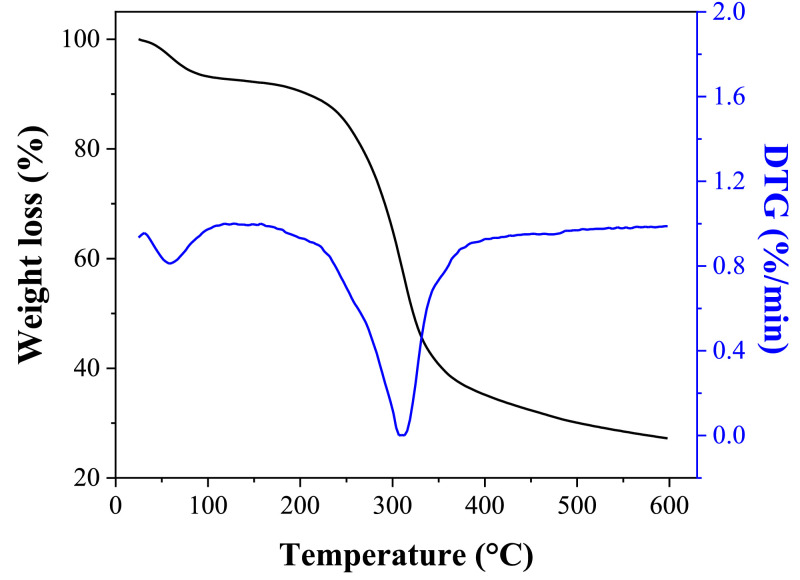
Weight loss percentage and its corresponding DTG of OPEFB fibers.

Moreover, the data from thermogravimetric analysis (TGA) in terms of thermal stability of OPEFB fibers are shown in Fig. 3., where four stages of weight loss were observed around the following temperature ranges: 25–150 °C, 150–250 °C, 250–350 °C, and 410–600 °C.

**Fig. 4:**
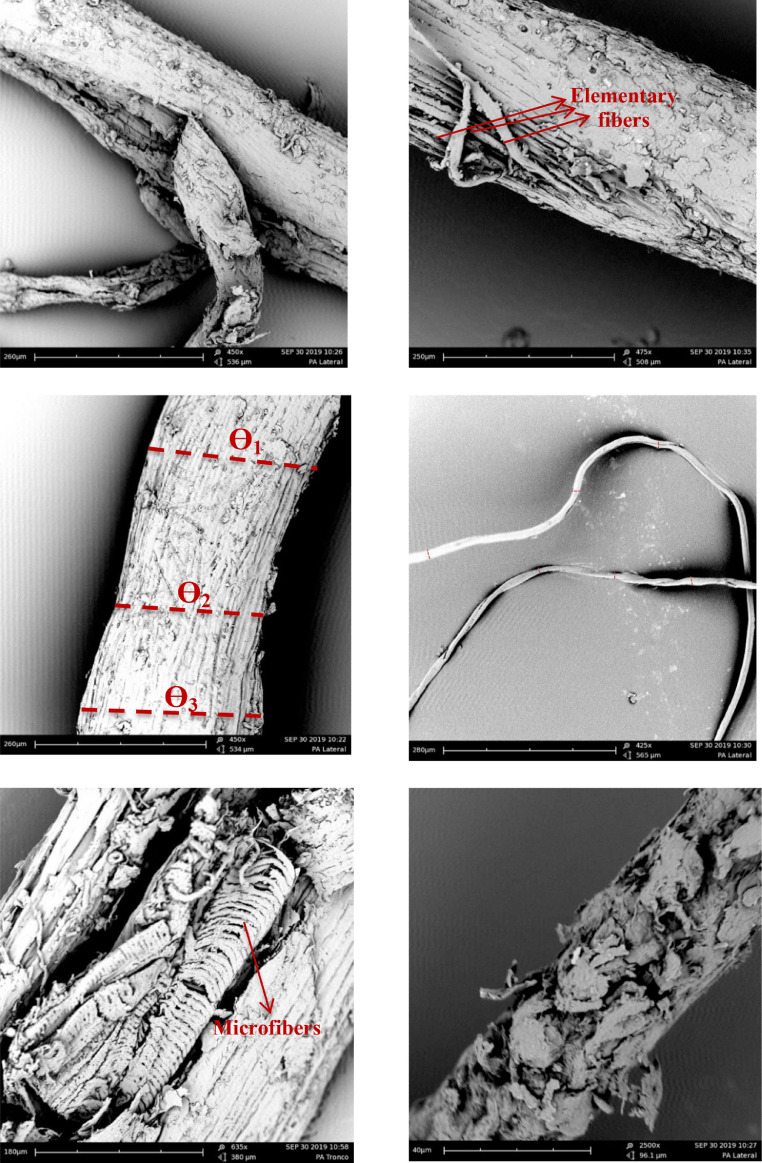
SEM images of the lateral section of OPEFB fibers with the presence of irregular/porous surface, heterogeneous diameter, elementary fibers, and microfibers. Ɵ= diameter.

Lastly, scanning electron microscopy (SEM) micrographs of OPEFB fibers at 425, 450, 475, 635, and 2500× magnification are displayed in Fig. 4. The fibers exhibited a porous surface and the presence of elementary fibers and microfibers. Additionally, a variation in diameter along fiber length was observed.

## 2 Experimental Design, Materials, and Methods

**Fig. 5:**
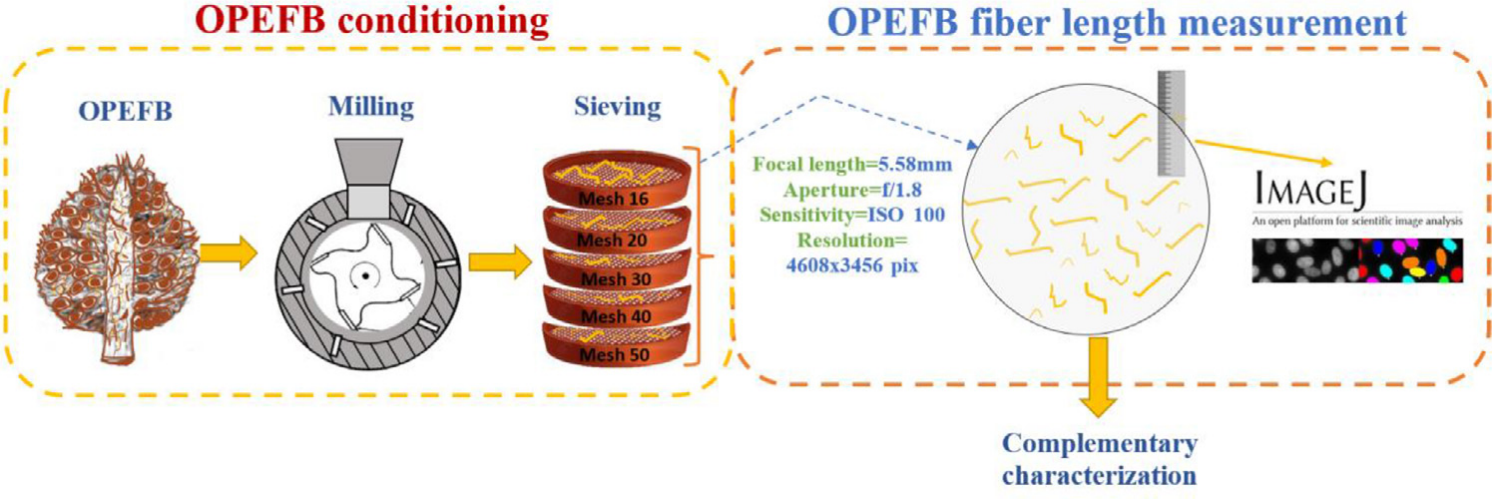
Methodology for conditioning OPEFB waste and measuring OPEFB fiber length.

OPEFB waste was collected from palm oil extraction industries located in the northwest of Ecuador. The waste was dried at room temperature, ground in a blade mill (SHINI model SG-2348E), and then classified by a sieve shaker (HUMBOLT model H-4325) and a set of ASTM laboratory analytical sieves of five aperture lengths (mesh No.: 16, 20, 30, 40, and 50) as can be seen in Fig. 5. Thereafter, the fiber length groups studied, which were dried separately at 103°C for 3 h, consisted of non-sieved OPEFB fibers and OPEFB fibers retained on meshes No. 20, 30, 40, and 50 due to unknown size limits related to retained fibers on mesh 16.

### 2.1 OPEFB Fiber Length Measurement

OPEFB fiber length was determined using a methodology that led to more data being collected in less time. Initially, about 40 photographs of OPEFB fibers were taken from each of the length groups mentioned above. The OPEFB fiber length measurement was carried out by processing the photographs with the ImageJ® software. Images were taken keeping focal length (5.58 mm), aperture (f/1.8), sensitivity (ISO 100), and resolution (4608×3456 pixels) constant. About 53,000 measurements were made for non-sieved OPEFB fibers, while 21,500, 17,800, 23,000, and 33,000 for OPEFB fibers retained on meshes No. 20, 30, 40, and 50, respectively. The measurement scale was established with a standard precision graduated ruler. The methodology for conditioning OPEFB waste and measuring the fiber length is depicted in Fig. 5.

### 2.2 Statistical Analysis

Statistical analysis of the distribution fit of length data was performed assuming that, during the grinding process of OPEFB waste, the fibers were randomly cut into smaller ones, and the number of these elements was expected to decrease exponentially with respect to their length; similar to what has been reported in other mechanical processes involving natural fibers [8], [9], [10]. In addition, it was assumed that a particular OPEFB fiber was randomly cut along its length by the blade mill, being (m) the average cuts per unit length and (ml) the average cuts in a fiber of length (l). In like manner, the number of cuts (n) for a particular fiber of length (l) was assumed to follow a Poisson distribution (Eq. (6)).

(6) $$f\left(n\right)=\frac{1}{n!}{\left(ml\right)}^{n}e^{-ml}$$

On the other hand, the probability of the event in which a fiber segment of length (l) was not cut into smaller fibers by the blade mill was considered to be:

(7) $$f\left(0\right)=e^{-ml}$$

Taking into account that the aforementioned probability was the same as the probability of obtaining a fiber longer than a length (l) after the grinding process, the cumulative distribution function for the fiber length was:

(8) $$F\left(l\right)=1-e^{-ml}$$

Then, the distribution for the fiber length was obtained by differentiating Eq. (8), as follows:

(9) $$f\left(l\right)=me^{-ml}$$

Considering the previously mentioned about (m) being the average cuts in a fiber per unit length, the average fiber length (θ) after the grinding process was:

(10) $$\theta =\frac{1}{m}$$

Consequently, the distribution for the fiber length became:

(11) $$f\left(l\right)=\frac{1}{\theta }e^{-\;\frac{l}{\theta }}$$

Finally, a minimum fiber length $\left(l_{0}\right)$ obtained from the grinding process was considered due to the blade mill features, such as the screen diameter. Therefore, the distribution for the fiber length (Eq. (11)) was converted into Eq. (1), where the variable $\left(l-l_{0}\right)$ followed an exponential distribution with mean (θ).

Likewise, since the gamma distribution is a generalization of the exponential distribution but with a shape parameter of (k=1), it was assumed that the length data of the sieved OPEFB fibers followed, in general, three-parameter gamma distributions, as detailed in Eq. (2).

All the distribution parameters, both for the exponential and for the gammas, were determined using MLE.

On the other hand, in the case of the two-parameter exponential distribution, the standard errors $\left(\sigma_{l_{0}}\right)$ and $\left(\sigma_{\theta }\right)$ were calculated considering the variances $Var(l_{0})$ and Var(θ) [11], which are presented in Eqs. (12) and (13).

(12) $$\sigma_{l_{0}\;}=\frac{\theta }{n}$$

(13) $$\sigma_{\theta }=\frac{\sqrt{n-1}}{n}\;\theta$$

where, the standard error $\left(\sigma_{l_{0}}\right)$ was inversely proportional to the number of observations, and the standard error $\left(\sigma_{\theta }\right)$ was approximately inversely proportional to the square root of the number of observations.

Whereas, for the three-parameter gamma distribution, the standard errors $\left(\sigma_{l_{0}}\right)$, $\left(\sigma_{k}\right)$, and $\left(\sigma_{\theta }\right)$ were calculated using the Fisher information matrix (I(k,θ)) presented in Eq. (14); from which, the variance of each parameter corresponded to each entry on the diagonal of the inverse matrix $\left(I^{-1}\right)$ [12].

(14) $$I\left(k,\theta \right)=n\left(\begin{array}{ccc} \frac{1}{\theta^{2}\left(k-2\right)} & \frac{1}{\theta \left(k-1\right)} & \frac{1}{\theta^{2}}\\ \frac{1}{\theta \left(k-1\right)} & \psi_{1}\left(k\right) & \frac{1}{\theta }\\ \frac{1}{\theta^{2}} & \frac{1}{\theta } & \frac{k}{\theta^{2}} \end{array}\right)$$

where, being the inverse matrix proportional to $\left(\frac{1}{n}\right)$, the standard errors (i.e., square root of variance) of each parameter were proportional to $\left(\frac{1}{\sqrt{n}}\right)$, e.g., doubling the number of observations reduced the error by a factor of $\sqrt{2}$.

All the standard errors of the distribution parameters, both for the exponential and for the gammas, were determined with the Python library SciPy® using the scipy.stats.expon and scipy.stats.gamma methods.

Moreover, the proposed theoretical distributions were tested against a myriad of other distributions using residual sum of squares (RSS) with the Python package distfit®; from which, the exponential and gamma distributions were the best fit to the OPEFB fiber length data.

To determine whether the fiber length data differed from the theoretical distributions obtained, the KS test was carried out considering a level of significance of 0.01. Nevertheless, since the original KS test is no longer valid when distribution parameters are directly estimated from data [13], [14], [15], a parametric bootstrap procedure was carried out to approximate the null distribution [16,17]. Being non-sieved fibers a mixture of lengths i.e., long and short fibers, the operator errors during length measurement increased, as well as the differences, but in much less intense, between the observations and the proposed theoretical distribution. However, since no other theoretical distribution validated by the KS test was found, it was assumed that there was no other better than the exponential distribution to model the length data of non-sieved fibers. Whilst, for sieved fibers retained on mesh No. 40 (for which, the p-value was lower than the assumed level of significance, and the best fit found was a generalized gamma distribution), it was considered appropriate to keep the three-parameter gamma distribution in order to have a general model [18].

### 2.3 Complementary Characterization

Chemical composition of OPEFB fibers was determined in order to quantify the content of lignin (ASTM D1106), hemicellulose/cellulose (ASTM D1109), and extractives (ASTM D1107-ASTM D1110). In addition, moisture (ASTM D4442) and ash (ASTM D1102) content were accomplished. The mechanical properties (ASTM D2256) of OPEFB fibers were carried out with a universal testing machine (INSTRON model 5544). A load cell of 100 N and a speed of 30 mm/min were used during the test. The mean values of Young's modulus, elongation at break, and toughness were determined using 30 specimens.

Infrared characteristics were obtained by FTIR spectroscopy in attenuated total reflection mode using a spectrometer (JASCO model FT/IR-C800). Twenty scans were completed in a range between 4000 and 600 cm^−1^ with a resolution of 4 cm^−1^. Furthermore, TGA was accomplished using a thermobalance (SHIMADZU model TGA-50). OPEFB fibers were heated at 10°C/min under a nitrogen flow of 50 mL/min. The mass loss (%) of OPEFB fibers was analyzed between 20 and 600 °C.

SEM was performed on an electronic microscope (FEI Phenom model FP3950/00). OPEFB fibers were fixed on metallic supports with carbon surfaces, and then coated with Au/Pd in an argon atmosphere for 135 s under a current of 18 mA using a Sputter Coater SC7620 (Quorum). The distance between the samples and the coating source was 35 mm. Afterwards, samples were placed in the SEM chamber, and micrographs were taken at different magnifications.

## Ethics Statements

Hereby, authors consciously assure that for the manuscript “Characterization dataset of oil palm empty fruit bunch (OPEFB) fibers – Natural reinforcement/filler for materials development”, the following is fulfilled:

• This material is the authors’ own original work, which has not been previously published elsewhere.
• The paper reflects the authors’ own research and analysis in a truthful and complete manner.
• The paper properly credits the meaningful contributions of co-authors and co-researchers.
• The data are appropriately placed in the context of prior and existing research.
• All sources used are properly disclosed (correct citation). Literally copying of text must be indicated as such by using quotation marks and giving proper reference.
• All authors have been personally and actively involved in substantial work leading to the paper, and will take public responsibility for its content.

We agree with the above statements and declare that this submission follows the policies of Data in Brief as outlined in the Guide for Authors and in the Ethical Statement.

## CRediT authorship contribution statement

**Alex Darío Aguilar:** Conceptualization, Methodology, Investigation, Writing – review & editing. **Vladimir Valle:** Conceptualization, Visualization, Writing – review & editing, Project administration, Funding acquisition. **Cristina E. Almeida-Naranjo:** Data curation, Writing – original draft. **Ángel Naranjo:** Formal analysis, Writing – review & editing. **Francisco Cadena:** Supervision, Visualization. **Jerónimo Kreiker:** Resources. **Belén Raggiotti:** Resources.

## Declaration of Competing Interest

The authors declare that they have no known competing financial interests or personal relationships that could have appeared to influence the work reported in this paper.

## Data Availability

Oil Palm Empty Fruit Bunch (OPEFB) Fiber Length (Original data) (Mendeley Data).
